# Supplementary material for: Socioeconomic status differences in psychological responses to unfair treatments: Behavioral evidence of a vicious cycle
Source: PLoS One. 2022 Jun 10;17(6):e0268286. doi: 10.1371/journal.pone.0268286 (PMC9187106; doi:10.1371/journal.pone.0268286)
Supplement: S1 File — (DOCX) [file pone.0268286.s001.docx]

# Supporting Information for

Socioeconomic status differences in psychological responses to unfair treatments:

Behavioral evidence of a vicious cycle

## **S1 Table**. Study 2: The results of the multiple regression analysis testing the effects of objective SES on perceived unfairness during the dictator game

| Predictors | Perceived unfairness during the dictator game | | | | | |
| --- | --- | --- | --- | --- | --- | --- |
|  | Model 1 | | | Model 2 | | |
|  | B | SE | 95% CI | B | SE | 95% CI |
| Objective social class | 0.205^†^ | .112 | [–0.015, 0.426] | 0.227^†^ | .121 | [–0.013, 0.467] |
| Gender |  |  |  | 0.041 | .180 | [–0.315, 0.397] |
| Age |  |  |  | –0.087^*^ | .034 | [–0.154, –0.019] |
| Political orientation |  |  |  | 0.034 | .071 | [–0.107, 0.175] |
| System justification belief |  |  |  | 0.060 | .138 | [–0.214, 0.334] |
| Financial insecurity |  |  |  | 0.050 | .066 | [–0.082, 0.181] |
| adjusted R^2^ | .018 | | | .034 | | |

Notes. Unstandardized coefficients are given; CI = confidence interval for B; Gender: 0 = men & 1 = women; ^†^ *p* < .1, ^*^ *p* < .05.


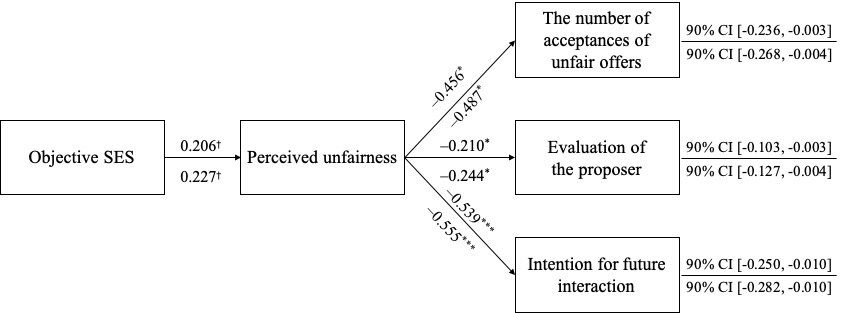


**S2 Figure.** Study 2: The results of the mediation analysis testing the indirect effects of objective SES via perceived unfairness on subsequent psychological responses. Unstandardized coefficients are given above (without covariates models) and below (with covariates models) the arrow/lines. 90% CI = 90% bootstrap confidence interval for indirect effects (Bootstrap Sample = 10,000). In case of objective SES, only 90% CI for indirect effects did not include zero; Total effects and direct effects parameters are reported in S3 Table; ^†^ *p* < .1, ^*^ *p* < .05, ^***^ *p* < .001.

## **S3 Table.** Study 2: The results from regression analyses testing mediation where objective SES is associated with DVs indirectly through perceived unfairness.

|  | Perceived unfairness | | The number of acceptances of unfair offers | | Evaluation of the proposer | | Intention for future interaction | |
| --- | --- | --- | --- | --- | --- | --- | --- | --- |
| Predictor | B(SE) | 95% CI | B(SE) | 95% CI | B(SE) | 95% CI | B(SE) | 95% CI |
| Objective SES |  |  |  |  |  |  |  |  |
| Total effect | 0.206^†^(.112) | [-0.015, 0.426] | 0.332(.248) | [-0.159, 0.822] | 0.018(.125) | [-0.231, 0.266] | -0.021(.183) | [-0.383, 0.341] |
| Direct effect |  |  | 0.425^†^(.247) | [-0.063, 0.914] | 0.061(.125) | [-0.187, 0.309] | 0.090(.176) | [-0.258, 0.437] |
| Perceived unfairness |  |  | -0.456^*^(.193) | [-0.837, -0.074] | -0.210^*^(.098) | [-0.404, -0.016] | -0.539^***^(.137) | [-0.811, -0.267] |
| Objective SES |  |  |  |  |  |  |  |  |
| Total effect | 0.227^†^(.121) | [-0.013, 0.467] | 0.454(.274) | [-0.089, 0.997] | 0.070(.139) | [-0.204, 0.344] | -0.180(.201) | [-0.577, 0.217] |
| Direct effect |  |  | 0.564^*^(.273) | [0.024, 1.104] | 0.125(.138) | [-0.148, 0.398] | -0.055(.192) | [-0.435, 0.326] |
| Perceived unfairness |  |  | -0.487^*^(.200) | [-0.883, -0.091] | -0.244^*^(.101) | [-0.444, -0.044] | -0.555^***^(.141) | [-0.834, -0.275] |
| Gender | 0.041(.180) | [-0.315, 0.397] | -0.232(.399) | [-1.021, 0.558] | 0.029(.201) | [-0.370, 0.428] | 0.371(.281) | [-0.186, 0.928] |
| Age | –0.087^*^(.034) | [-0.154, -0.019] | -0.042(.077) | [-0.195, 0.111] | -0.044(.039) | [-0.121, 0.033] | -0.022(.055) | [-0.130, 0.086] |
| Political orientation | 0.034(.071) | [-0.107, 0.175] | 0.095(.158) | [-0.218, 0.408] | -0.009(.080) | [-0.167, 0.149] | -0.037(.112) | [-0.258, 0.183] |
| System justification belief | 0.060(.138) | [-0.214, 0.334] | 0.277(.307) | [-0.331, 0.885] | 0.218(.155) | [-0.089, 0.524] | 0.098(.217) | [-0.331, 0.526] |
| Financial insecurity | 0.050(.066) | [-0.082, 0.181] | 0.227(.148) | [-0.066, 0.519] | 0.130^†^(.075) | [-0.017, 0.278] | -0.142(.104) | [-0.348, 0.064] |

Notes. Unstandardized coefficients are given; CI = confidence interval for B; Gender: 0 = men & 1 = women; ^†^ *p* < .1, ^*^ *p* < .05, ^***^ *p* < .001.

## **S4 Table**. Study 2: The results from regression analyses testing mediation where subjective SES is associated with DVs indirectly through perceived unfairness.

|  | Perceived unfairness | | The number of acceptances of unfair offers | | Evaluation of the proposer | | Intention for future interaction | |
| --- | --- | --- | --- | --- | --- | --- | --- | --- |
| Predictor | B(SE) | 95% CI | B(SE) | 95% CI | B(SE) | 95% CI | B(SE) | 95% CI |
| Subjective SES |  |  |  |  |  |  |  |  |
| Total effect | 0.291^*^(.120) | [0.053, 0.529] | 0.315(.270) | [-0.219, 0.849] | 0.057(.136) | [-0.212, 0.327] | 0.072(.199) | [-0.321, 0.466] |
| Direct effect |  |  | 0.452(.271) | [-0.084, 0.988] | 0.121(.137) | [-0.151, 0.393] | 0.236(.192) | [-0.144, 0.616] |
| Perceived unfairness |  |  | -0.470^*^(.195) | [-0.856, -0.085] | -0.220^*^(.099) | [-0.416, -0.025] | -0.563^***^(.138) | [-0.836, -0.290] |
| Subjective SES |  |  |  |  |  |  |  |  |
| Total effect | 0.376^**^(.132) | [0.115, 0.637] | 0.439(.304) | [-0.164, 1.042] | 0.126(.153) | [-0.177, 0.429] | -0.082(.223) | [-0.523, 0.358] |
| Direct effect |  |  | 0.635^*^(.308) | [0.027, 1.244] | 0.226(.154) | [-0.080, 0.532] | 0.137(.217) | [-0.292, 0.566] |
| Perceived unfairness |  |  | -0.523^*^(.204) | [-0.926, -0.120] | -0.266^*^(.102) | [-0.468, -0.063] | -0.584^***^(.143) | [-0.868, -0.300] |
| Gender | 0.074(.177) | [-0.275, 0.424] | -0.164(.399) | [-0.954, 0.626] | 0.050(.201) | [-0.347, 0.447] | 0.378(.281) | [-0.179, 0.934] |
| Age | -0.095^**^(.034) | [-0.162, -0.029] | -0.059(.078) | [-0.214, 0.095] | -0.051(.039) | [-0.129, 0.026] | -0.029(.055) | [-0.138, 0.080] |
| Political orientation | 0.057(.071) | [-0.083, 0.197] | 0.130(.160) | [-0.187, 0.446] | 0.006(.080) | [-0.153, 0.165] | -0.024(.113) | [-0.247, 0.199] |
| System justification belief | 0.043(.136) | [-0.226, 0.313] | 0.259(.307) | [-0.350, 0.868] | 0.209(.154) | [-0.097, 0.514] | 0.087(.217) | [-0.341, 0.516] |
| Financial insecurity | 0.072(.065) | [-0.057, 0.202] | 0.231(.148) | [-0.063, 0.524] | 0.146(.075) | [-0.002, 0.293] | -0.108(.105) | [-0.315, 0.099] |

Notes. Unstandardized coefficients are given; CI = confidence interval for B; Gender: 0 = men & 1 = women; ^*^ *p* < .05, ^**^ *p* < .01, ^***^ *p* < .001

## **S5 Table**. Study 1: The results from regression analysis testing the effects of family income on perceived unfairness

| Predictors | Perceived unfairness during the dictator game | | | |
| --- | --- | --- | --- | --- |
|  | B | SE | *p* value | 95% CI |
| Family income | 0.0009 | 0.0003 | .004 | [0.0003, 0.0015] |
| Gender | –0.0981 | 0.1511 | .517 | [–0.3954, 0.1992] |
| Age | –0.0075 | 0.0096 | .437 | [–0.0264, 0.0114] |
| Political orientation | –0.0001 | 0.0630 | .999 | [–0.1240, 0.1239] |
| System justification belief | –0.1142 | 0.0959 | .234 | [–0.3028, 0.0745] |
| Financial insecurity | 0.0780 | 0.0480 | .105 | [–0.0165, 0.1724] |

*Note.* Unstandardized coefficients are given; CI = confidence interval for B; Gender: 0 = men & 1 = women
