# Supplementary material for: Socioeconomic status differences in psychological responses to unfair treatments: Behavioral evidence of a vicious cycle
Source: PLoS One. 2022 Jun 10;17(6):e0268286. doi: 10.1371/journal.pone.0268286 (PMC9187106; doi:10.1371/journal.pone.0268286)
Supplement: Attachment — Submitted filename: review2022.docx [file pone.0268286.s002.docx]

The author used two studies to explore the difference of higher and lower socioeconomic status (SES) during unfair treatments. The topic is potentially of interest, however, as detailed below, the manuscript in its current form has several structures, academic expression, experimental design, and statistics shortcomings that need to be addressed.

**Introduction**

1, Line 65, who are the other people (Kim et al., 2021)? Please clarified.

2, The relationship between perceive unfair treatments and tolerance for unfairness should be clarified. The authors addressed that “lower SES individuals—would perceive unfair treatments as less problematic than higher SES individuals (Line 80-81)”, and then the downstream consequences are differences in one’s tolerance for unfair treatments (Line 83-84). However, in Line 95-97, the authors illustrated that “…how SES differences in initial tolerance for unfairness could have downstream consequences…”. This sentence means that initial tolerance for unfairness is ealier than perceive unfair treatments. Likewise, in introduction, authors proposed that the first goal is to test the prediction “lower SES individuals would perceive unfair treatments as less problematic than higher SES individuals”. However, in “The Present Research” section, authors proposed that the first goal is to test “lower SES individuals would be more tolerant to unfair treatments than higher SES individuals”. Perceive unfair treatments and tolerance for unfairness are distinctive term.

3, the difference between DG and UG should be addressed with more details.

**Study 1**

1, in the Introduction, the authors identified one’s tolerance for unfair treatments as the likely to take an action against the unfair treatment (Line 85-86). However, in the study 1, the authors suggested that “participants indicated how unfair the offers from the proposer were (1 = *not at all* to 7 = *a great deal; M* = 5.75, *SD* = 1.34) as an index of tolerance for unfair treatments.” (Line 178-179). This measurment is related to “perceive unfair treatments”, rather than tolerance for unfair treatments. Moreover, the first sentence in Study 1 also mentioned that the aim of the Study 1 is to related to perceive unfair.

2, It is not clear whether SES groups mean the subjective SES categories. Moreover, 95% CI should be reported regarding the correlations. I suggested that the regression analysis is more suitable to reveal the relations between subjective SES classes and perceived unfairness, with gender, age, political orientation, system justification belief, and financial insecurity as covariations.

**Study 2**

1, Line 322, “indicating that lower SES individuals tended to be more tolerant to apparent unfair distributions during the DG” also confused the tolerant to apparent unfair distributions and perceived apparent unfair distributions.

**Discussion**

1, The authors can further illustrate that how their data contribute on the theories of fairness.
