# Supplementary material for: Socioeconomic status differences in psychological responses to unfair treatments: Behavioral evidence of a vicious cycle
Source: PLoS One. 2022 Jun 10;17(6):e0268286. doi: 10.1371/journal.pone.0268286 (PMC9187106; doi:10.1371/journal.pone.0268286)
Supplement: Attachment — Submitted filename: response to reviewers.docx [file pone.0268286.s003.docx]

# Editor

## E1.

First, I find the SES categorization strange. More parsimonious would be to look just at income, which, in S1, does correlate with perceived unfairness (making lines 193-194 incorrect. Income correlated positively with perceived unfairness (r = .118, p = .032). Why not just use this basic analysis. It seems very robust to covariate inclusion and doesn’t involve any subjective judgments about what constitutes high vs. low SES.

First of all, we would like to thank you for this thoughtful comment. We agree with you that income is one of the most important indicators of one’s SES and it may be better to use income without worrying about any subjective judgements. However, SES is such a multifaceted concept (e.g., [Kraus & Stephens, 2012](https://doi.org/10.1111/j.1751-9004.2012.00453.x)) and there may be some aspect of SES that income may not be able to capture. In particular, income itself may not determine one’s social status in Confucian cultures like Korea. Therefore, we wanted to include other indicators of SES (e.g., education or job prestige) than income in the present research.

In addition, Study 1 selectively recruited participants belonging to either higher SES or lower SES based on our criteria specified in the main text. In other words, participants in the higher SES and lower SES groups in Study 1 can be said to be quite distinctive in terms of income, education, and job prestige. Therefore, we think that it may be better to do a group comparison (as we did in the present manuscript) than to treat SES as a continuous variable using income (please see also our response to E2).

Nevertheless, we do think that it can be informative as well as interesting to run the basic analyses you suggested in your review. Therefore, following your comment, we run the suggested analyses using income as an indicator of SES. As shown in the table below, the analyses led us to the same conclusion as the main analyses we reported in the main text. In the present revision, we reported the results in the Supporting Information (S5 Table). We also added a footnote in the main text.

*(Footnote 2 on Page 12):*

Among the three SES criteria, income was positively correlated with perceived unfairness (*r* = .118, *p* = .032). We repeated the same analysis using income as an indicator of SES and reached the same conclusion (see S5 Table).

## E2.

Related to this, I’m surprised that subjective SES doesn’t correlate with the DV of interest. Typically, in psychology, we find that subjective assessments of dimensions like wealth or SES have a great influence on judgments and behavior than objective dimensions do. Why do you believe that, in this case, that doesn’t hold?

It is indeed intriguing to see that subjective SES does not correlate with perceived unfairness, while income does show a significant correlation with perceived unfairness in Study 1. As you correctly pointed out, subjective SES tend to show stronger associations with psychological processes than objective SES in the relevant literature. Thus, the finding is somewhat inconsistent with the previous findings. However, we were reluctant to elaborate such dissociation in the present revision. First, the finding was not replicated in Study 2. If there is anything, subjective SES showed slightly stronger associations with our DVs of interest than objective SES in Study 2 (as in the previous studies).

Moreover, as we briefly mentioned in our response to E1, we intended to create higher vs. lower SES group in Study 1. Specifically, individuals were assigned to the lower SES group when they have less than a BA degree (educational level), less than the median household income in Korea (income), and a temporary/blue-collar job (occupation). In contrast, individuals were assigned to the higher SES group when they have BA or more advanced degree, more than the median household income in Korea, and permanent/white-collar jobs. In other words, we believe that we created two group that are quite distinctive in terms of their SES. Thus, in our opinion, it may be more appropriate to analyze and interpret differences between the higher and lower SES groups than to treat SES as a continuous variable based on objective and subjective SES.

However, we think that it is worthwhile to elaborate the difference between objective and subjective SES. Therefore, in response to your comment, we included a relevant discussion on this issue in the present revision.

*(Lines 448 – 468 on Pages 24-25):*

In the present research, we included several measures of SES covering both subjective and objective aspects of SES. Specifically, participants indicated educational attainment (personal and parental education in Studies 1 & 2, respectively), family income, and subjective perception of their SES. Thus, we conducted additional analyses in order to explore which indicator of SES would show a particularly strong association with perceived unfairness. In Study 1, income was positively associated with perceived unfairness, *r* = .118, *p* = .032 and the association became marginal for educational attainment, *r* = .100, *p* = .071. Curiously, subjective SES was not correlated with perceived unfairness, *r* = .036, *p* = .512. Thus, the results in Study 1 are somewhat inconsistent with the previous studies suggesting that subjective construal of one’s rank in the social hierarchy would be a more powerful predictor of social cognitive tendencies than objective indices (e.g., Kraus et al., 2012). However, in our opinion, such inconsistency should be cautiously interpreted. First, Study 1 recruited two groups that were quite distinctive in terms of their SES (i.e., education, income, and occupation). Thus, it may not be appropriate to treat SES as a continuous variable as in the correlational analyses. Rather, it may be better to conduct a group comparison as we reported in the main results. Second and more importantly, the results were not replicated in Study 2. In Study 2, subjective SES showed a stronger association than objective SES (i.e., income and education) as in the previous literature, Subjective SES: *r* = .209, *p* = .017, Income: *r* = .142, *p* = .107, & Parental education: *r* = .114, *p* = .198. Therefore, we think that future research should systematically investigate the potential differences between subjective and objective SES in psychological processes reported in the present research.

## E3.

Line 225 "The inclusion of 225 these participants did not change the direction of the results.” This statement, though true, is pretty misleading. In fact, the critical relationship between perceived SES and fairness drops well below the conventional levels of significance when including all participants. Indeed, for the excluded participants, there is a NEGATIVE relationship between SES and fairness (r = -.29, p = .10). It’s not clear to me why you would expect to see this, nor why this exclusion criteria is justified. Mere familiarity with the games shouldn't fundamentally flip the relationship between the key IV and DV. What rationale do you provide for this and was this exclusion criteria pre=determined and pre-registered, or was it made once the results were known?

We excluded participants who were familiar with the resource allocation games used in the present research (i.e., the dictator and ultimatum games) and yet, did not state exactly why decided to do so. The reason why we excluded them is that students typically learn about rational choices in this kind of resource allocation games during the several courses in economics or social/cognitive psychology. For example, given that a recipient ends up with nothing when rejecting a given offer, it is usually considered as rational to accept even the smallest possible offer in the ultimatum game (UG) from a purely economic standpoint. Thus, participants with this kind of knowledge may be willing to accept smaller offers than those without such knowledge. In fact, Carter and Irons (1991) found that the amount of acceptable offer in the UG was smaller for students majoring in economics than students in other disciplines. Based on this-related findings, we decided to exclude participants who are familiar with the resource allocation games used in the present research. In the present revision, we included our rationale for the exclusion and noted that the inclusion of those participants weakened the results.

*(Lines 248 – 253 on Page 13):*

Thirty-two of them were excluded due to their familiarity with both resource allocation games (i.e., DG & UG) since previous knowledge about these games is shown to systematically influence one’s responses such that knowledgeable participants tend to accept even the smallest possible reward in the UG as it is considered as rational from an economic perspective (e.g., Carter & Irons, 1991). The inclusion of these participants did not change the direction of the results although the effects became substantially weaker.

By the way, we were not aware that the association between SES and perceived fairness is reversed among those excluded participants, as you found. At this point, we do not have any good explanation for the finding. Given that even modest amount of offer may be perceived as reward among them due to their knowledge on the UG, we speculate that approach tendency on the part of higher SES participants (e.g., [Keltner et al., 2003](https://doi.org/10.1037/0033-295X.110.2.265)) may make them accept smaller offer than lower SES participants. However, this interpretation is too speculative and thus, we did not include it in the present research.

## E4.

In Study 2, like with S1, I’m surprised that actual income wasn’t used as a key predictor of fairness. In this study, family income is only weakly correlated with fairness (r = .14, p - .11) for the sub-sample and completely uncorrelated for the full sample (r = .07, p = .36). Again, this feels like a much more natural IV, and yet it is largely ignored.

As you pointed out, it may seem weird not to use household income as a predictor in our main analyses. However, please note that our participants in Study 2 were college students and there are some ambiguities in using household income among college students. First, students may not be aware of their household income and thus, their report may not be accurate. In addition, some college students in Korea live with their parents while others live independently. In our opinion, this is another reason why household income may not be a good indicator of one’s SES among college students. Also, we reason that this kind of ambiguities may contribute to the null/weak effect of household income in Study 2.

## E5.

Beyond this, there the review team feels as though there are valid questions about the use of covariates (R1), the structure of the results for S2 can be cleaned up (R1), there can be more reporting of key statistics (R1 and R2), there is confusion about the results for S2 (specifically as it relates to Figure 2 and Table 3; R1), there is concern about the use of exclusively students in S2 (R1), and, overall, the language throughout the paper could use some more precision (R1 and R1).

We agree with you that both reviewers raised valid and interesting questions. We addressed them in our responses to each reviewer below.

# Reviewer 1

**Major comments**

## R1-1.

1. The Introduction (page 3, line 37, 38) rather strongly reports that lower SES is more likely to be treated unfairly. From line 46 onwards, it is explained a bit further. Although I agree, but I still think that the unfair treatment should be explained further? How does the unfair treatment look like exactly? Perhaps some readers might not know why some treatments are unfair and why low SES receive these more often. What people consider unfair differs between people (also within socioeconomic groups).

In response to this point, we provided further explanation about unfair treatments in the present revision.

*(Lines 34 – 38 on Page 3):*

In addition, such awareness is particularly important among individuals with lower socioeconomic status (SES) as they face societal/personal unfairness more often than individuals with higher SES due to financial/educational constraints, low social capital, and negative stereotypes/ discrimination (Laurin et al., 2019).

*(Lines 50 – 55 on Pages 3-4):*

For example, lower SES individuals are treated more unfairly than higher SES individuals in a way that can reinforce the existing social hierarchy (Magee & Galinsky, 2008; Schminke et al., 2000). Similarly, lower SES individuals are frequent targets of negative stereotyping and discrimination (e.g., Batruch et al., 2017; Darley & Gross (1983; Stephens et al., 2007). For example, Rivera and Tilcsik (2016) found that higher SES applicants were more successful than lower SES applicants in spite of identical credentials.

## R1-2.

2. Financial insecurity is considered a confounder: it is not fully clear. I would consider it more likely a mediator or an alternative measure of SES. In any of those cases, controlling for financial insecurity might lead to overadjusted (underestimated) association of SES with the outcomes.

First of all, we agree with the reviewer that financial insecurity could be treated as a mediator or an alternative measure of SES. However, we decided to treat it as a covariate in the present research for the following reason. In the present research, we would like to show that lower SES individuals would end up developing tolerance for unfair treatments due to the repeated exposure to unfair treatments. In other words, it is their mindset (rather than financial difficulty) that would lead lower SES individuals to accept a small amount of monetary reward regardless of how unfair it is. Toward this end, we included financial insecurity as a control variable in order to (at least partially) rule out an alternative explanation that lower SES individuals would be more willing to accept even unfair monetary offers than higher SES individuals only because of their financial difficulty. In the present revision, we further clarified why we decided to use financial insecurity as a control variable.

*(Lines 174 - 181 on Page 10):*

Similarly, we hypothesized that lower SES individuals would show a habitual/psychological tendency to tolerate unfair treatments and yet, financially insecurity itself (regardless of psychological tendencies) may force one to accept even unfair offers. Thus, we included the In-Charge Financial Distress/Financial Well-Being Scale (Prawitz et al., 2006) that measures the degree of financial insecurity across eight items (1 = *not at all* to 10 = *a great deal*; Cronbach’s α = .927; *M* = 6.19, *SD* = 1.82). in order to (at least partially) rule out an alternative hypothesis that lower SES individuals would be more willing to accept even unfair monetary offers than higher SES individuals only because of their financial difficulty.

In addition, please note that we found the same results with or without controlling for financial insecurity. As we reported in the main text, one’s SES was associated with perceived unfairness regardless of financial insecurity (Study 1: *F* (1, 320) = 4.79, *p* = .029; Study 2: *b* = 0.325, *SE* = 0.124, *p* = .010). Furthermore, the indirect effects in Study 2 remained the same even without including financial insecurity. Please see below.

**Table.** Indirect effect of Subjective SES on DV in Study 2 without including financial insecurity

|  | Outcome variables | | |
| --- | --- | --- | --- |
| Predictor | The number of acceptances of unfair offers | Evaluation of the proposer | Intention for future interaction |
| Subjective SES | 95%CI | 95%CI | 95%CI |
|  | [–0.3606, –0.0199] | [–0.1775, –0.0145] | [–0.4016, –0.0461] |

*Notes*. 95% CI = 95% bootstrap confidence interval for indirect effects (Bootstrap Sample = 10,000).

## R1-3.

3. Are the Results of Study 2 not too elaborate? Reading the research questions, one is waiting until line 348 and onwards where the answers follow, particularly Figure 2. All the previous is very much secondary. The same holds for several of the additional outcomes. The main one in the UG is "number of acceptances of unfair offers". All others are secondary (evaluation of proposer and intention for future interaction). Try to concentrate on the main findings and help readers to see where the primary focus should be.

As this reviewer pointed out, we also think that Figure 2, in particular results regarding number of acceptances in UG, is the most important finding in Study 2. However, we conceptualized the findings in Figure 2 as downstream consequences of perceived unfairness. Therefore, we think that the association between SES and perceived unfairness is the first domino to fall. In addition, although auxiliary, we think that descriptive statistics such as binary correlations could be quite informative. Thus, in the present revision, we presented the results in the same order as the previous manuscript. Instead, we clearly emphasized the important findings in Study 2

*(Lines 374 – 376 on Page 21):*

In this section, we addressed the most important questions in Study 2 regarding behavioral and interpersonal consequences of the SES differences in perceived unfairness.

## R1-4.

4. Table 3 indicates that SES (1 and 2) is related to perceived unfairness (3), but not to the number of acceptances of unfair offers (6). Figure 2, however, shows that there is an association between SES and the number of acceptances of unfair offers (which is mediated by the perceived unfairness). This is really puzzling. If it is caused by some kind of suppressor effects, please clarify.

As this reviewer correctly pointed out, SES was associated with the number of acceptance of unfair offers indirectly through perceived unfairness and yet, the simple association between SES and the number of acceptance was not statistically significant. Significant indirect effect in the absence of total effect should be carefully interpreted. In the present revision, we first clearly descried that we only found significant indirect effect and then, carefully discussed its implications.

*(Lines 395 – 396 on Page 22):*

In other words, subjective SES itself was not significantly associated with DVs (see S4 Table for detailed results).

*(Lines 475 – 479 on Pages 25-26):*

This suggests that SES may not be inherently associated with passive reactions. Rather, SES may be associated with various factors that have dynamic relations with passive reactions. Thus, future research should investigate other factors (than perception of unfair treatments) that play an important role in the relation between SES and psychological responses to unfairness.

In addition, several researchers have argued that a significant indirect effect can exist even in the absence of total/direct effects (see Hayes, 2016; Rucker et al., 2011; Zhao et al., 2010). Moreover, we would like to note that it is not uncommon in the literature to report the indirect effect of social class/SES without the significant total effects as in the present research (e.g., [Sommet et al., 2019](https://onlinelibrary.wiley.com/doi/10.1111/jopy.12432); [Belmi et al., 2020](https://doi.org/10.1037/pspi0000187)).

**Minor comments**

## R1-5.

1. Political orientation (1 very liberal and 7 very conservative) is supposed to correlate with acceptance of unfair treatment. What is the hypothesized direction of that association (and why?)?

We did not have any specific hypothesis regarding political orientation. However, we included it as a control variable since political conservatism is associated with maintain status quo and justification of inequality (e.g., [Jost et al., 2003](https://doi.org/10.1037/0033-2909.129.3.339)), which, in turn, may be associated with the tendency to accept unfair offers. .

## R1-6.

2. Study 2 are all students: is the variation in the key determinant, i.e. SES, not restricted by this selection?

We agree with the reviewer that the generalizability of Study 2 is limited because we only recruited college students. However, we would like to note that we managed to recruit socioeconomically diverse participants in Study 1. Also, the restriction of variations in SES works against our predictions, which make the present research a conservative test of our predictions. In fact, this restriction in sample may explain why subjective SES showed stronger associations than objective SES such as income in Study 2 (please also see our response to E4). In the present revision, we addressed this issue in the discussion section.

*(Lines 479 – 484 on Page 26):*

Additionally, our participants may not be diverse enough. For example, participants in Study 2 were college students and thus, there may not be sufficient variations in SES among them. Although we recruited non-college samples and found similar results in Study 1, all of our participants were Koreans. Therefore, the generalizability of the current findings should be further investigated in other cultural contexts with more demographic diversity.

## R1-7.

3. The unfair offers in both Study 1 and Study 2 should be explained a bit further (preferably not using more words). In Figure 1 for example, it is unclear that the proposer gets 26 and the recipient 4 (experimenter announces) and then there are 3 rounds. It is just unclear, explain super-clearly (it is fascinating to know exactly how the unfairness is created).

In response to this point, we tried to provide a clearer description about our experimental procedures. We also modified Figure 1 on page 14.

*(Lines 189 -193 on Page 10):*

Specifically, participants received 3, 1, & 0 coins, whereas the proposer kept 7, 9, & 10 coins in the first, second, and third round, respectively (i.e., 1R = 3:7, 2R = 1:9, & 3R = 0:10). To emphasize unfairness, participants were informed that the proposer obtained 26 (7+9+10) coins, whereas they obtained a total of 4 (3+1+0) coins across the three rounds.

## R1-8.

4. When rejecting, both proposer and recipient get zero coins (line 263): would knowing that imply that one needs to control for additional variables (e.g. solidarity, empathy)? Why are the participants informed like that?

Following the standard protocol in the Ultimatum game, we informed participants that both proposer and recipient would get nothing when the recipient reject the offer from the proposer. As this reviewer pointed out, this procedure may influence how the recipient behave in the UG. Initially, we had the same concern but we found that lower SES individuals behaved similarly in the UG regardless of whether the rejection would lead to zero reward or not (e.g., [Ding et al., 2017](http://dx.doi.org/10.1016/j.jesp.2017.03.008)). Based on the finding, we decided to use the standard instruction of the Ultimatum game.

## R1-9.

5. Check carefully, I saw several instances of plural ("are") where it should be singular ("is) (e.g. line 424, 401).

First, we would like to thank the reviewer for their careful reading of the manuscript. In response to this point, we corrected this kind of typos in the present revision.

# Reviewer 2

**Introduction**

## R2-1.

1, Line 65, who are the other people (Kim et al., 2021)? Please clarified.

In response to this point, we made the following change in the present revision.

*(Line 67-69 on Page 4):*

For example, lower SES individuals are less optimistic regarding their future (Boehm et al., 2015), and they have lower trust in other people in general (Kim et al., 2022) than higher SES individuals.

## R2-2.

2, The relationship between perceive unfair treatments and tolerance for unfairness should be clarified. The authors addressed that “lower SES individuals—would perceive unfair treatments as less problematic than higher SES individuals (Line 80-81)”, and then the downstream consequences are differences in one’s tolerance for unfair treatments (Line 83-84). However, in Line 95-97, the authors illustrated that “…how SES differences in initial tolerance for unfairness could have downstream consequences…”. This sentence means that initial tolerance for unfairness is earlier than perceive unfair treatments. Likewise, in introduction, authors proposed that the first goal is to test the prediction “lower SES individuals would perceive unfair treatments as less problematic than higher SES individuals”. However, in “The Present Research” section, authors proposed that the first goal is to test “lower SES individuals would be more tolerant to unfair treatments than higher SES individuals”. Perceive unfair treatments and tolerance for unfairness are distinctive term.

First of all, we agree with the reviewer that we should have clearly mentioned the relationship between perceived unfairness and tolerance for unfairness. We reason that, if one perceives apparent unfair treatments as *less* unfair than others, the person can be said to be relatively tolerant of unfair treatments, compared to others. That is, in our reasoning, tolerance for unfairness is equivalent to perceiving unfair treatments as less unfair. Accordingly, we used perceived unfairness and tolerance for unfairness interchangeably in the present manuscript. This may be confusing, as the reviewer pointed out. Therefore, we clearly described what we meant by tolerance for unfairness (on page 6) and made several revisions throughout the present revision. If it is still confusing, then we are more than willing to make further changes (e.g., not using the term, tolerance for unfairness) in the next round of revision:

*(Lines 118 – 123 on Page 6):*

Specifically, in Study 1, we investigated the relationship between SES and tolerance for unfairness by measuring how lower and higher SES participants perceived a series of unfair offers they had to accept during the DG. Here, we operationalized the tolerance for unfairness in relation to the perception of unfair treatment. We reason that, if one perceives apparent unfair treatments as less unfair than others, the person can be said to be relatively tolerant of unfair treatments, compared to others.

## R2-3.

3, the difference between DG and UG should be addressed with more details.

In response to this point, we provided more explanation regarding the difference between the Dictator Game (DG) and the Ultimatum Game (UG) in the present revision.

*(Lines 108 – 116 on Page 6):*

Both games are an anonymous two-person decision game involving a proposer and a recipient. In the DG, a proposer (i.e., a dictator) decides on a split of monetary resources. Then, a recipient remains passive and has to accept the proposer’s allocation. In the UG, a proposer suggests a split of monetary resources to the recipient, similar to the DG. However, unlikely the DG, the recipient can choose to either accept or reject the proposer’s offer. If the recipient accepts, both players receive the proposed allocation. If the recipient rejects the offer, neither player receives anything. In other words, if the proposer decides how to distribute allocated resources between them, the recipient should accept the proposals in the DG and yet, the recipient can reject the proposals in the UG.

**Study 1**

## R2-4.

1, in the Introduction, the authors identified one’s tolerance for unfair treatments as the likely to take an action against the unfair treatment (Line 85-86). However, in the study 1, the authors suggested that “participants indicated how unfair the offers from the proposer were (1 = *not at all* to 7 = *a great deal; M* = 5.75, *SD* = 1.34) as an index of tolerance for unfair treatments.” (Line 178-179). This measurement is related to “perceive unfair treatments”, rather than tolerance for unfair treatments. Moreover, the first sentence in Study 1 also mentioned that the aim of the Study 1 is to related to perceive unfair.

As we said in our response to R2-2, if individuals perceive unfair treatments as less unfair, we believe that they are tolerant of unfairness. That is, perception of unfairness is critically linked to tolerance for unfairness in the present research and thus, we use them interchangeably. In order to clarify this, we made several changes in the present revision (please see our response to R2-2).

## R2-5.

2, It is not clear whether SES groups mean the subjective SES categories. [R2-5a] Moreover, 95% CI should be reported regarding the correlations. [R2-5b] I suggested that the regression analysis is more suitable to reveal the relations between subjective SES classes and perceived unfairness, with gender, age, political orientation, system justification belief, and financial insecurity as covariations. [R2-5c]

[R2-5a] In Study 1, we recruited two distinctive SES groups (i.e., the higher SES group and the lower SES group) based on one’s education, income, and job prestige. Less than college-educated temporary/blue-collar workers with less than median income were assigned to the lower SES group, whereas white-collar workers with BA or more advanced degrees and more than median income were assigned to the higher SES group. This group division is what we meant by the SES group (please also see our responses to E1 & E2). We made this clear in the current revision

*(Lines 155 -158 on Page 8):*

In Study 1, we targeted lower and higher SES samples based on objective criteria. We used three objective criteria most frequently used in the relevant literature (e.g., Kraus & Stephen, 2012) to recruit participants with lower vs. higher SES backgrounds:

*(Lines 198 - 199 on Page 11):*

Using this subjective measure of SES, we examined whether our division of higher SES vs. lower SES groups were valid.

[R2-5b] Following your comment, we reported 95% CI for the correlation coefficients between all measures in Table 2 (for Study 1) and Table 3 (for Study 2).

[R2-5c] Given that what we are essentially doing in Study 1 is a two-group comparison (i.e., higher SES vs. lower SES groups), we believe that an independent sample *t*-test and an ANCOVA with covariates should provide the same results as a regression analysis with and without control variables. In fact, as shown in the table below, the regression analysis proposed by the reviewer showed the essentially the same result as the ANCOVA analysis reported in the main text. In the present revision, we decided to report an independent *t*-test and an ANCOVA rather than the equivalent regression analyses since we believe that group means and SDs are more intuitive than regression coefficients. However, if you and the reviewer think otherwise, we will reconsider this decision.

**Table**. The results of multiple regression analysis testing the effects of SES group on perceived unfairness during the dictator game (Study 1)

| Predictors |  | Perceived unfairness during the dictator game | | | | |
| --- | --- | --- | --- | --- | --- | --- |
|  | B | | SE | *t* | *p* value | 95% CI |
| SES group | 0.4218 | | 0.1614 | 2.61 | .009 | [0.1042, 0.7393] |
| Gender | –0.0559 | | 0.1518 | –0.37 | .713 | [–0.3546, 0.2428] |
| Age | –0.0048 | | 0.0096 | –0.50 | .619 | [–0.0238, 0.0142] |
| Political orientation | 0.0048 | | 0.0632 | 0.08 | .939 | [–0.1194, 0.1291] |
| System justification belief | –0.1182 | | 0.0961 | –1.23 | .220 | [–0.3073, 0.0709] |
| Financial insecurity | 0.0731 | | 0.0482 | 1.52 | .130 | [–0.0217, 0.1680] |

*Notes*. SES group: 0 = lower SES group & 1 = higher SES group; Gender: 0 = men & 1 = women.

**Study 2**

## R2-6.

1, Line 322, “indicating that lower SES individuals tended to be more tolerant to apparent unfair distributions during the DG” also confused the tolerant to apparent unfair distributions and perceived apparent unfair distributions.

Again, in our conceptualization, tolerance for unfairness is equivalent to perceiving unfair treatments as less unfair. Thus, to the extent that a given participant perceived one-sidedly disadvantageous offers as less unfair than others, the participant can be considered as relatively more tolerant to unfair treatments than others in the present research. Please also refer to our responses to R2-2 & R2-4.

**Discussion**

## R2-7.

1, The authors can further illustrate that how their data contribute on the theories of fairness.

In response to this point, we made several revisions in the discussion section by addressing this-related issues. In particular, we added the following paragraph in the present revision

*(Lines 419 – 428 on Page 23):*

A substantial body of literature on fairness and equality has demonstrated that individuals generally show inequality aversion (e.g., Bolton, 1991), and thus most people reject unfairness even at the expense of personal resources (Fehr & Schmidt, 1999). Moreover, since disadvantageous people are more likely to be on the receiving end of societal injustice (compared with advantageous people), lower SES people have a greater incentive to be sensitive (and react) to the unfair situation. However, across two studies, we found that lower SES participants perceived unfair treatment as less unfair compared with higher SES participants. These findings may indicate that the lower (vs. higher) SES individuals have a different baseline of unfairness/fairness such that lower SES individuals are more tolerant of unfairness than higher SES individuals.
